# Time to act: Fast -Tracking the response for people who inject drugs in Asia and the Pacific

**DOI:** 10.1186/s12954-015-0077-7

**Published:** 2015-10-16

**Authors:** Steve Kraus, Jeremy Douglas

**Affiliations:** UNAIDS Regional Support Team for Asia and the Pacific, United Nations Building, Rajadamnern Nok Avenue, Bangkok, 10200 Thailand; UNODC for Southeast Asia and the Pacific, United Nations Building, Rajadamnern Nok Avenue, Bangkok, 10200 Thailand

## Commentary

Asia and the Pacific region is one of the world’s most vibrant places in terms of economic growth and social advancement. It captures many superlatives: the planet’s highest proportion of young people, the largest mega-cities in the world and Asia wins the crown of "global growth leader" again and again. However, despite the dynamism, some citizens are falling through the cracks. This is particularly true for people who inject drugs (PWID). With 3.15 million PWID, in terms of sheer numbers, East Asia and Southeast Asia has the largest injecting drug problem in the world and is home to one in four PWID globally [1].

People who inject drugs have multiple vulnerabilities—to HIV, hepatitis, tuberculosis and other infectious diseases; they account for an estimated 30 % of new HIV infections outside sub-Saharan Africa. About a third of PWID are living with HIV in Southwest Asia [1], and a number of countries have reported HIV prevalence higher than 10 % [2]. National figures often do not tell the full story, with some localities reporting significantly higher prevalence. In Cebu City, Philippines, HIV prevalence among PWID was estimated at 52.3 % in 2013 [3], and in Faisalabad, Pakistan, it was as high as 52.5 % in 2011 [4].

Scientific evidence shows that to respond to HIV and other health risks of people who inject drugs, it is important to develop programmes which cater to their needs. The Joint United Nations Programme on HIV/AIDS (UNAIDS), the United Nations Office on Drugs and Crime (UNODC) and the World Health Organization (WHO) have recommended a package of nine interventions, commonly referred to as the comprehensive approach to injecting drug use [5].

The first four interventions—needle-syringe programmes (NSP), opioid substitution therapy (OST), HIV testing and counselling and the provision of antiretroviral therapy (ART)—are critical for effective national programmes. There is compelling evidence that NSP and OST are effective in reducing the sharing of injecting equipment and averting HIV infections. In combination with ART, these interventions diminish HIV transmission, decrease mortality, reduce drug dependency and improve quality of life.

Several countries in the region have taken these recommendations to heart and implemented parts of the comprehensive harm reduction package. In the late 1990s, China had a fast-growing HIV epidemic among people who inject drugs and its health authorities began introducing OST sites and needle-syringe programmes. By the end of 2014, more than 700 OST sites were operating in China. HIV incidence rate dropped by 87.6 % from 0.95 % in 2006 to 0.12 % in 2014 among those registered in the clinics [6]. Recently, India and Myanmar have also increased their OST coverage.

Despite these pockets of success, the coverage of harm reduction programmes remains inadequate in Asia and the Pacific region. High coverage of needle-syringe programmes is defined by WHO, UNAIDS and UNODC as more than 200 needles or syringes provided per PWID a year. Only four countries reported reaching high coverage in 2014, and five reported that less than 50 % of PWID used clean equipment for their last injection [2].

PWID frequently face stigma and discrimination when seeking health services and are far less likely to receive ART than others living with HIV due to social marginalization and the flawed perception that they are unable to adhere to treatment effectively. While current national monitoring systems are not designed to capture data on access to ART for PWID, a recent review suggests that less than five PWID received ART per 100 HIV-positive PWID [7].

Not only is coverage low, but there are worrying signs that some countries with a high HIV burden among PWID are scaling down harm reduction interventions [8]. This is undoubtedly due to the phasing out of support by international donors with little new funds coming in from domestic sources. In Asia and the Pacific, 81 % of harm reduction activities were funded by international partners, primarily the Global Fund to Fight AIDS, Tuberculosis and Malaria and key bilateral partners [9].

Criminalization and punitive policies are often an entrenched part of the drug rehabilitation landscape in Asia and the Pacific region. The most common response is the confinement of people who use drugs in compulsory treatment and rehabilitation centres. In many centres, a medical assessment of drug dependency is not undertaken upon admission and evidence-informed treatment is lacking for those with drug dependency. Where treatment is provided, it is often not efficient, resulting in high relapse rates upon release from the centres.

In March 2012, 12 UN agencies issued a joint statement on compulsory drug detention and rehabilitation centres, calling for their closure and replacement with voluntary, evidence-informed and rights-based health and social services in the community [10]. Malaysia has taken the lead in making the change by transforming 22 compulsory detention centres for PWID into ‘Cure and Care’ centres and establishing 10 clinics that provide outpatient treatment [11].

Yet this move to voluntary community based treatment and services is still the exception rather than the rule. A paradigm shift is needed, moving from a punitive approach to support, praise and encouragement. With the world’s biggest population of PWID, the Asia-Pacific region can demonstrate the entrepreneurial can-do spirit it is known for and lead the way to ending the AIDS epidemic among people who inject drugs.

To achieve this, there is an urgent need to rapidly scale up evidence-informed services for PWID in the next 5 years. This fast-track approach sets the following targets to be reached by 2020:

- Ninety percent of PWID know their HIV status
- Ninety percent of PWID who know their status are receiving treatment
- Ninety percent of PWID on treatment have a suppressed viral load so their immune systems remain strong
- Ninety percent coverage of HIV prevention services for PWID
- Forty percent of PWID access opioid substitution therapy
- Zero discrimination towards PWID

Countries should also make an effort to increase access to naloxone through community-based distribution to help reduce high rates of opioid overdose, particularly where access to essential health services for PWID is limited. In addition, access to hepatitis B and C prevention, screening and treatment services should be accelerated.

While these targets are ambitious, they are possible to reach if countries:

- Replace compulsory drug detention and rehabilitation centres with voluntary and community-based treatment and services in a time-bound transition plan developed with key stakeholders
- Increase domestic investment in a comprehensive package of prevention, care and treatment services for PWID
- Strengthen collaboration between drug control and law enforcement officials and the health sector and update legal and policy frameworks to allow the expansion of HIV services to PWID
- Work on the intersection between injecting drug use and high-risk sexual behaviour, especially where sex work is undertaken to buy drugs or accessed by men who have sex with men, sex workers and transgender people

This approach is transformative. If business as usual continues, it is estimated that there will be around 20,000 new HIV infections among PWID in the Asia and Pacific region by 2030, but with a Fast-Track approach that number could be cut by more than half. These numbers speak for themselves. The time to act is now. The region has achieved much, and we are confident that with visionary leadership, solid partnerships between government and civil society, evidence-informed and targeted allocation of resources and bold action, it can become the first in the world to end the AIDS epidemic in PWID.
